# Cardiac Telocytes and Fibroblasts in Primary Culture: Different Morphologies and Immunophenotypes

**DOI:** 10.1371/journal.pone.0115991

**Published:** 2015-02-18

**Authors:** Yihua Bei, Qiulian Zhou, Siyi Fu, Dongchao Lv, Ping Chen, Yuanyuan Chen, Fei Wang, Junjie Xiao

**Affiliations:** 1 Regeneration and Ageing Lab, Experimental Center of Life Sciences and Innovative Drug Research Center, School of Life Science, Shanghai University, Shanghai 200444, China; 2 Shanghai Key Laboratory of Bio-Energy Crops, School of Life Science, Shanghai University, Shanghai 200444, China; 3 Division of Gastroenterology and Hepatology, Digestive Disease Institute, Tongji Hospital, Tongji University School of Medicine, Shanghai 200065, China; Centro Cardiologico Monzino, ITALY

## Abstract

Telocytes (TCs) are a peculiar type of interstitial cells with very long prolongations termed telopodes. TCs have previously been identified in different anatomic structures of the heart, and have also been isolated and cultured from heart tissues in vitro. TCs and fibroblasts, both located in the interstitial spaces of the heart, have different morphologies and functionality. However, other than microscopic observation, a reliable means to make differential diagnosis of cardiac TCs from fibroblasts remains unclear. In the present study, we isolated and cultured cardiac TCs and fibroblasts from heart tissues, and observed their different morphological features and immunophenotypes in primary culture. Morphologically, TCs had extremely long and thin telopodes with moniliform aspect, stretched away from cell bodies, while cell processes of fibroblasts were short, thick and cone shaped. Furthermore, cardiac TCs were positive for CD34/c-kit, CD34/vimentin, and CD34/PDGFR-β, while fibroblasts were only vimentin and PDGFR-β positive. In addition, TCs were also different from pericytes as TCs were CD34 positive and α-SMA weak positive while pericytes were CD34 negative but α-SMA positive. Besides that, we also showed cardiac TCs were homogenously positive for mesenchymal marker CD29 but negative for hematopoietic marker CD45, indicating that TCs could be a source of cardiac mesenchymal cells. The differences in morphological features and immunophenotypes between TCs and fibroblasts will provide more compelling evidence to differentiate cardiac TCs from fibroblasts.

## Introduction

Telocytes (TCs), a distinctive type of interstitial cells, have firstly been identified by Popescu’s group [1]. TCs are characterized by a small cell body and extremely long and thin telopodes with alternation of dilations (podoms) and thin segments (podomeres) [2–7]. The presence of TCs has been demonstrated in almost all mammalian organs and tissues [8–26]. For example, TCs have been identified in different anatomic structures of the heart, including epicardium [2], endocardium [3], myocardium [20,27], and heart valves [28]. Also, TCs in primary culture from heart tissues have been documented [29,30]. It was proved that TC could form a three-dimensional (3D) interstitial network by connecting with a variety of cells [28,29,31–37], and might play potential but important roles in tissue homeostasis [27,38,39], intercellular communication [4,29,35,37] and tissue repair/remodeling [40–47].

To date, transmission electron microscopy (TEM) remains a golden standard method for identifying TCs, since not a single immunomarker can be considered specific for detecting TCs [48]. However, to use a set of double immunolabeling for TCs is of fundamental importance since it allows to explicit their presence and to distinguish them from other types of interstitial cells [49,50]. Discrimination between TCs and fibroblasts is an important subject in TC identification. In theory, distinction between TCs and fibroblasts is evident as they have different morphological characteristics and functions [48,51]. Moreover, differences of gene profiles [52,53], microRNA signatures [54], as well as proteome features [55] between TCs and fibroblasts have been described in heart or lung tissues. Morphologically, cell processes of fibroblasts are usually few, short and thick, which make it theoretically easy to distinguish them from TCs under light microscope. However, some of the cells tagged as “fibroblasts” in the literature have similar morphological features as TCs, thus probably leading to confusion when identifying these two cell types [48].

The aim of the present study was to observe different morphological features of cardiac TCs and fibroblasts in primary culture, and to determine their different immunophenotypes by using a set of double immunofluorescent staining for CD34/c-kit, CD34/vimentin, and CD34/PDGFR-β, which might provide more reliable means and evidence to discriminate between TCs and fibroblasts in heart tissues.

## Materials and Methods

### Animals

Male C57BL/6 mice, weighed 25–30 g (10–12 weeks), were purchased from the animal research center of Fudan University. Mice were housed in a temperature-controlled room on a 12 h light/dark cycle, with *ad libitum* access to food and water. All animal experiments were conducted under the guidelines on the use and care of laboratory animals for biomedical research published by National Institutes of Health (No. 85–23, revised 1996). This study was approved by the committee on the Ethics of Animal Experiments of Tongji Hospital (Permit Number: KYSB-2014–41). All surgery was perfomed under sodium pentobarbital anesthesia and all efforts have been made to mininmize suffering.

### Isolation and primary culture of TCs and fibroblasts from heart tissues

After mice were anesthetized with 1% pentobarbital sodium, the hearts were isolated under sterile conditions and kept in Hank’s balanced salt solution (HBSS, R21–022-CV, Corning, NY, USA), supplemented with 100 U/ml penicillin and 100 μg/ml streptomycin (R30–002-CI, Corning) and 0.01 mM HEPES (H3375, Sigma, St. Louis, MO, USA). After transported to the cell culture room and rinsed again with fresh HBSS, the hearts were transferred into a sterile culture dish containing DMEM/F12 (12400–024, Gibco, New York, USA) supplemented with 0.25 mg/ml collagenase typeⅡ (17101–015, Invitrogen, Paisley, Renfrewshire, UK) and 0.01 mM HEPES. The hearts were then minced into 1 mm^3^ pieces, transferred into 50 mL centrifuge tube, and incubated on an orbital shaker at 37°C for 35 min with the collagenase solution as described above. After 25 mL ice-cold HBSS was added into the digest to inhibit collagenase activity, dispersed cells were separated from non-digested tissues by passing through a 40-μm-diameter cell strainer and collected with centrifugation at 1000 rpm for 5 min at 4°C. After washed once by centrifugation in HBSS, cells were resuspended with 10 mL DMEM/F12 supplemented with 10% fetal bovine serum (FBS, 16000–044, Gibco), 100 U/ml penicillin, and 100 μg/ml streptomycin, and seeded into sterile culture dishes. Cells were then cultured in a humidified atmosphere of 5% CO_2_ at 37°C for 2 h to purify the cell suspension with TCs by allowing fibroblasts attachment. The unattached cells (containing TCs) were collected and cultured in DMEM/F12 supplemented with 10% FBS, 100 U/ml penicillin, and 100 μg/ml streptomycin for 24 h and culture medium was changed. Cell cultures were examined using inverted biological microscope (BM-37XC, China), and TCs and fibroblasts were photographed under 200× magnification at 48 h, 72 h and 96 h after seeded.

### Isolation and primary culture of pericytes from heart tissues

Pericytes were isolated and cultured as previously described [56]. Briefly, mice cardiac tissues freed from atria and upper portions of the ventricles were minced in HBSS, and treated with 0.25% trypsin in DMEM at 37°C for 15 min, followed by digestion in 0.1% collagenase type II, 0.05% deoxyribonuclease, and 25 μM CaCl_2_ until soft. After DMEM containing 1% BSA and 100μM CaCl_2_ was added into the digest to inhibit collagenase activity, dispersed cells were separated by 100-μm-diameter cell strainer and collected with centrifugation at 1500 rpm for 5 min at 4°C. After washed with DMEM containing 1% BSA and 500μM CaCl_2_ and layered over 5ml of DMEM containing 6% BSA, cells were centrifugated at 800 rpm for 10 min to separat a clear upper layer from a darker layer. Cells from the latter were cultured in DMEM/F12 supplemented with 10% FBS, 100 U/ml penicillin, and 100 μg/ml streptomycin.

### Isolation and primary culture of bone marrow-derived mesenchymal stem cells (BMSC)

BMSCs were isolated and cultured as our previous described [57]. Briefly, primary BMSCs were harvested from tibias and femurs of 4-week-old Sprague-Dawley rats under aseptic conditions and then purified and passaged in DMEM/F12 supplemented with 10% fetal bovine serum, 100 U/ml penicillin, and 100 μg/ml streptomycin.

### Double immunofluorescent staining for CD34/c-kit or vimentin or PDGFR-β or α-SMA in cell cultures

After washed with PBS for three times, cells were fixed in 4% paraformaldehyde for 30 min, washed with PBS, and permeabilized with 0.5% Triton X-100 for 30 min. Cells were then washed with PBS and blocked in 3% bovine serum albumin (BSA) for 1 h. After that, cells were incubated overnight at 4°C with rat monoclonal anti-CD34 (ab8158, Abcam, Cambridge, MA, USA) and rabbit polyclonal anti-c-kit (ab5506, Abcam) primary antibodies diluted by 1:100 in 1% BSA. After washed with PBS for three times, cells were incubated with goat anti-rat FITC-labelled (sc-2011, Santa Cruz, CA, USA) and goat anti-rabbit rhodamine-labelled (E031320, Earthox, San Francisco, CA, USA) secondary antibodies diluted by 1:200 in 3% BSA for 2 h, and then stained with DAPI (F36924, Life technology, Grand Island, NY, USA). Finally, cells were kept in fresh PBS at 4°C in dark before observation. The images were taken under a magnification of 200× with fluorescent inverted microscope (Leica DMI4000 B, Germany). Similar procedures were used for double immunofluorescent staining for CD34/vimentin (rabbit monoclonal anti-vimentin; 2707–1, Epitomics, Burlingame, CA, USA) or CD34/PDGFR-β (rabbit monoclonal anti-PDGFR-β; ab32570, Abcam) or CD34/α-SMA (rabbit polyclonal anti-α-SMA; ab137734, Abcam).

### Flow cytometry analysis for cardiac TCs and BMSC

Cells were harvested by trypsinization and washed twice with cold PBS. TCs were stained with allophycocyanin-conjugated anti-mouse CD29 (17–0291, Ebioscence) and CD45 (17–0451, Ebioscence) while Rat-BMSCs were stained with FITC-conjugated anti-rat CD29 (561796, BD biosciences) and CD45 (561867, BD biosciences). As negative controls, cell aliquots were incubated with PBS under the same conditions. After incubation away from light for 30 min at 4°C, cells were analyzed by MoFlo XDP (Beckman Coulter) and FlowJo software (Tree Star).

## Results

### Different morphological features of cardiac TCs and fibroblasts in primary culture

**Fig 1:**
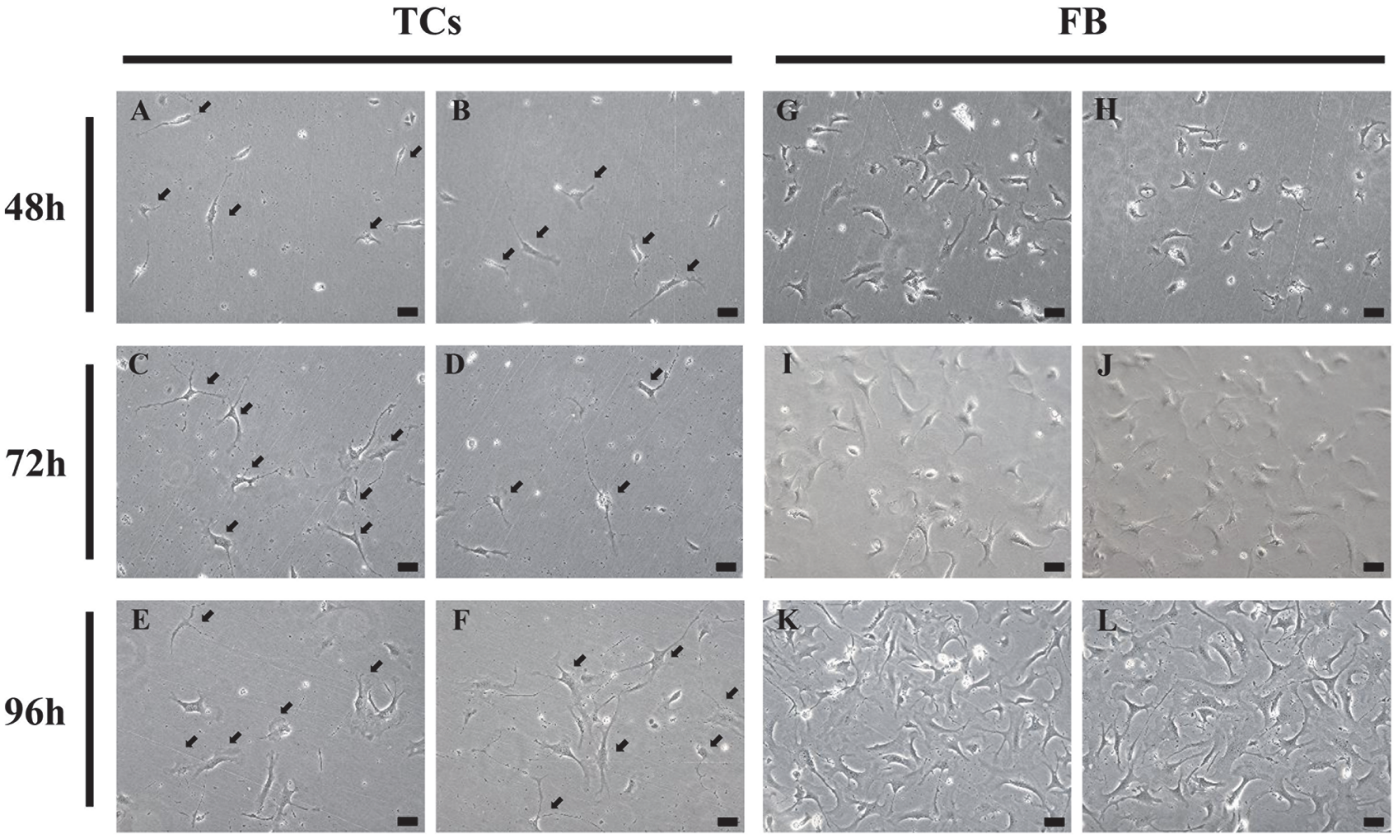
Light microscope shows cardiac telocytes (TCs, A-F) and fibroblasts (FB, G-L) in primary culture from heart tissues at 48 h, 72 h, and 96 h after the day of primary culture. Arrows show typical TCs with long and thin telopodes in primary culture. Original magnification 200×; Scale bar = 50 μm.

**Fig 2:**
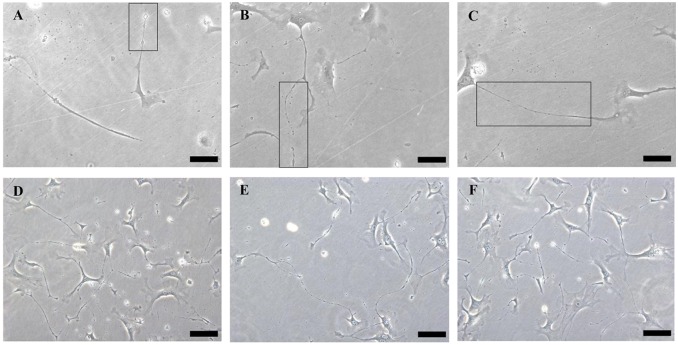
Light microscope shows telocytes (TCs) with typical morphological features. **(A)** A TC with small cell body and extremely thin and long telopode (Tp). **(B)** and **(C)** A TC with significant moniliform aspect: alternation of podoms-podomeres (enlarged in inset). **(D-F)** show more TCs with typical morphological features. Original magnification 200×; Scale bar = 50 μm.

Cardiac TCs in primary culture were purified from fibroblasts by using differential adhesion method. In the present study, fibroblast attachment occurred within 2 h after seeded, while TC attachment took 24 h. To observe the different morphological features of both cell types, cardiac TCs and fibroblasts were observed under light microscope and photographed at 48 h, 72 h and 96 h from the day of primary culture. As shown in Fig. 1, the prolongations of fibroblasts were relatively short, thick and cone shaped, while those of TCs were extremely long and thin, stretched away from cell bodies. Fig. 2 shows TCs with typical characteristics: small cell body and extremely thin and long telopode, and significant moniliform aspect with many dilations along telopode.

### Different immunophenotypes of cardiac TCs and fibroblasts in primary culture

**Fig 3:**
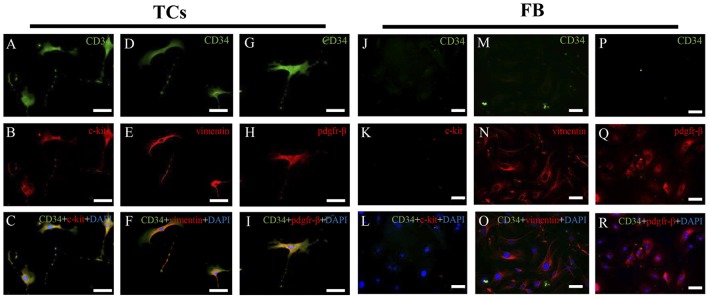
Double immunofluorescent staining for CD34/c-kit, CD34/vimentin and CD34/PDGFR-β of cardiac telocytes (TCs) and fibroblasts (FB) in primary culture. Fluorescent inverted microscope shows that TCs are positive for **(A)** CD34 (green) and **(B)** c-kit (red), while FBs are negative for **(J)** CD34 (green) and **(K)** c-kit (red). Co-localization of c-kit and CD34 (yellow) is significant in TCs **(C)**, while absent in FBs **(L)**. TCs are positive for **(D)** CD34 (green) and **(E)** vimentin (red). FBs are negative for **(M)** CD34 (green), but positive for **(N)** vimentin (red). Co-localization of vimentin and CD34 (yellow) is significant in TCs **(F)**, while absent in FBs **(O)**. TCs are positive for **(G)** CD34 (green) and **(H)** PDGFR-β (red). FBs are negative for **(P)** CD34 (green), but positive for **(Q)** PDGFR-β (red). Co-localization of PDGFR-β and CD34 (yellow) is significant in TCs **(I)**, while absent in FBs **(R)**. Nuclei are counterstained with DAPI (blue). Original magnification 200×; Scale bar = 50 μm. BF, bright field.

**Fig 4:**
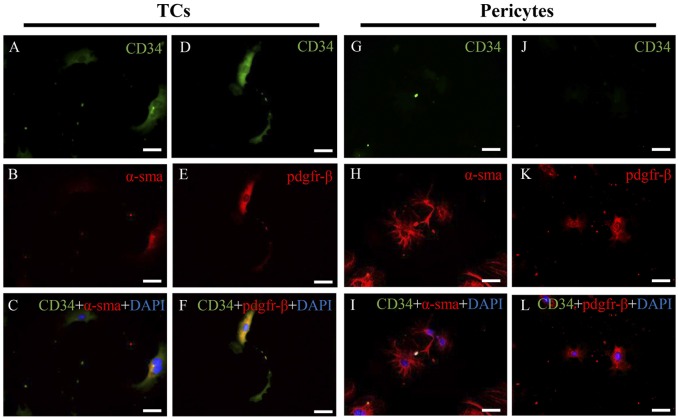
Double immunofluorescent staining for CD34/α-SMA and CD34/PDGFR-β of cardiac telocytes (TCs) and pericytes in primary culture. Fluorescent inverted microscope shows that TCs are positive for **(A)** CD34 (green) and relatively weak positive for **(B)** α-SMA (red), while pericytes are negative for **(G)** CD34 (green) but strong positive for **(H)** PDGFR-β (red). Co-localization of α-SMA and CD34 (yellow) is extremely weak in TCs **(C)**, while absent in pericytes **(I)**. TCs are positive for **(D)** CD34 (green) and **(E)** PDGFR-β (red), while pericytes are negative for **(J)** CD34 (green), but positive for **(K)** PDGFR-β (red). Co-localization of PDGFR-β and CD34 (yellow) was significant in TCs **(F)**, while absent in pericytes **(L)**. Nuclei were counterstained with DAPI (blue). Original magnification 200×; Scale bar = 50 μm. BF, bright field.

To identify different immunophenotypes of cardiac TCs and fibroblasts, three different double immunofluorescent methods (CD34 and c-kit, CD34 and vimentin, and CD34 and PDGFR-β) were used. As shown in Fig. 3, cardiac TCs were CD34/c-kit, CD34/vimentin and CD34/PDGFR-β positive, while cardiac fibroblasts were only vimentin and PDGFR-β positive, thus clearly demonstrating the different immunophenotypes of cardiac TC and fibroblasts in primary culture. As CD34 negative and vimentin positive cells in the culture may not all be fibroblasts, they could also be pericytes. We stained the fibroblast used in the present study and they were α-SMA negative (data not shown), indicating they were not pericytes. To futher compare the immunophenotypes of cardiac TCs and pericytes, we isolated pericytes and compared different immunophenotypes of cardiac TCs and fibroblasts. As indicated in Fig. 4, TCs were CD34 positive and α-SMA weak positive while pericytes were CD34 negative but α-SMA positive. In addition, TCs were CD34/PDGFR-β positive while pericytes were CD34 negative but PDGFR-β positive. These data indicated that TCs were different from pericytes.

### Cell surface markers of cardiac telocytes (TCs) and BMSC

**Fig 5:**
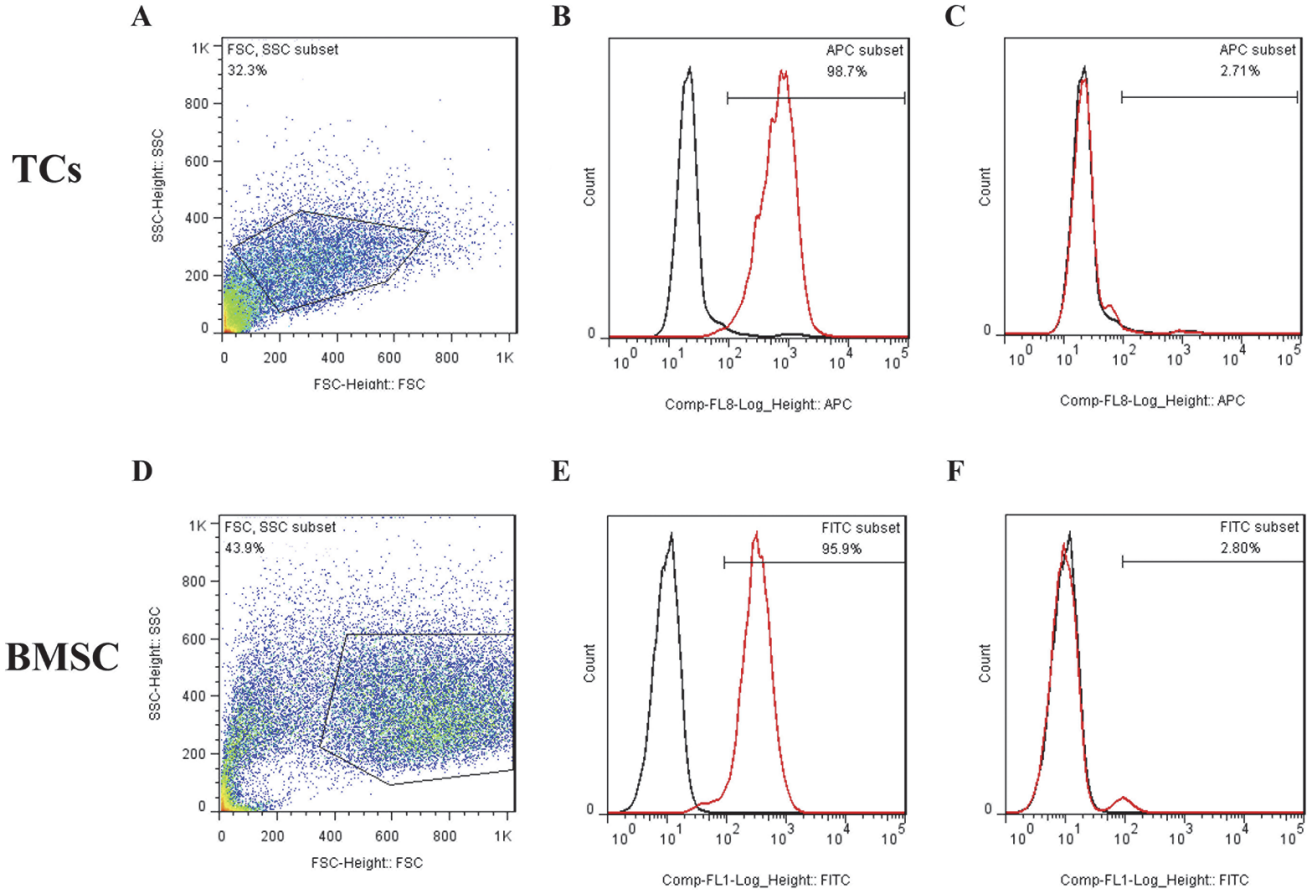
Cell surface markers of cardiac telocytes (TCs) and bone marrow-derived mesenchymal stem cells (BMSC). Cells collected are shown in A and D. Flow cytometry analysis show that cardiac TCs are homogenously positive for mesenchymal marker CD 29 (B) as BMSC (E), but negative for hematopoietic marker CD45 (C) as BMSC (F). Unlabeled cell (black) controls are included for comparison. FSC: forward scatter; SSC,side scatter.

As shown in Fig. 5, flow cytometry analysis showed that cardiac TCs were homogenously positive for mesenchymal marker CD29 but negative for hematopoietic marker CD45, which is similar to BMSC. These data indicate that TCs could be a source of cardiac mesenchymal cells, supporting that and they might hold the potential to give rise to MSCs in culture.

## Discussion

Cardiac TC and fibroblasts were isolated from heart tissues and their different morphological features were observed in primary culture. Meanwhile, we provided further evidence showing that cardiac TCs were positive for CD34/c-kit, CD34/vimentin, and CD34/PDGFR-β, while fibroblasts were only positive for vimentin and PDGFR-β, which clearly differentiated cardiac TCs from fibroblasts in primary culture.

Isolation and culture of TCs from a given organ is of great interest to study the peculiar morphological features and functionality of TCs [6,9–11,29,30,32,33,58]. TCs and fibroblasts, both located in the interstitium spaces, have different morphologies and functions [51]. Fibroblasts are mainly responsible for the production of collagen and some other extracellular matrix, whereas TCs are more functionally involved in intercellular communication via 3D network [51]. However, some cells labelled with “fibroblasts” or “fibroblast-like cells” in the literature are actually not real fibroblasts [48]. Thus, it is highly needed to discriminate between TCs and fibroblasts in virtual research. In the present study, we purified cardiac TCs from fibroblasts according to their different attachment time in primary culture. Actually, fibroblasts attached to culture dishes in less than 2 h after seeded, while cardiac TC attachment took much longer (2–24 h). When observed at 48 h, 72 h, and 96 h of primary culture, cell processes of fibroblasts were short, thick and cone shaped. While TCs extended very long and thin telopodes from cell bodies from 48 h of primary culture, coordinating with previous study [30]. Noteworthy, the alternation podoms-podomeres along telopodes clearly showed another typical morphological features of TCs.

Double immunolabeling is of great importance to make differential diagnosis of TCs from other cells in primary cultures or tissues [2,13,59]. In the present study, we performed double immunofluorescent staining for CD34/c-kit, CD34/vimentin, and CD34/PDGFR-β, which showed that cardiac TCs were positive for CD34, c-kit, vimentin and PDGFR-β, whereas fibroblasts were only positive for vimentin and PDGFR-β. Interestingly, similar to previous reports that not all TCs were CD34 and c-kit double positive [60], in TCs we isolated here, the proportion of CD34/c-kit double positive was 68.9%. It has previously been shown that pre-plating to remove fibroblasts from primary culture is not sufficient to completely clear fibroblasts, and that fibroblasts in primary culture express strongly vimentin but no CD34 [13,29]. However, immunolabeling for CD34 in combination with c-kit or vimentin still remains the best available approach to identify TCs from other cells [48]. In addition, PDGFR-β was also found positively expressed by TCs in heart valve, lung, skeletal muscle and liver samples [12,28,40,61,62]. The fibroblasts we isolated here were α-SMA negative, excluding the possibility of pericytes. Besides that, we also futher compared the immunophenotypes of TCs and pericytes. We found that TCs were CD34 positive and α-SMA weak positive while pericytes were CD34 negative but α-SMA positive, indicating that TCs were also different from pericytes. Anyway, the different immunophenotypes of cardiac TCs and fibroblasts by using double immunolabeling for CD34/c-kit, CD34/vimentin, and CD34/PDGFR-β, provides more compelling evidence to discriminate between cardiac TCs and fibroblasts.

It has been indicated that TCs could be a source of cardiac mesenchymal cells [63]. To address this point, using flow cytometry analysis, we showed that TCs were homogenously positive for mesenchymal marker CD29 but negative for hematopoietic marker CD45, which is similar to BMSC, supporting that they might hold the potential to give rise to MSCs in culture. In addition, as CD34+ cells may lose CD34 expression and acquire other marker expressions “in vivo” and “in vitro” [63], further studies over a longer period of culture to investigate whether the phenotype of the CD34+ cardiac TCs follows this behaviour.

In conclusion, the present study shows the different morphological features and immunophenotypes between cardiac TCs and fibroblasts in primary culture. Our results present here, as well as the comparison of gene profiles, microRNAs signatures and proteome features of these two cell types highly desirable to be determined in the future, will provide new lines of evidence to differentiate TCs from fibroblasts in heart tissues.
